# Diagnostic accuracy of gray-scale analysis on B-mode ultrasound for identifying intraplaque hemorrhage and lipid-rich necrotic core in carotid plaques

**DOI:** 10.1177/1358863X251410527

**Published:** 2026-02-25

**Authors:** Benjamin Wagner, Sasha Mukhija, Mohamed Kassem, Andrea Wiencierz, Mandy D Müller, Henrik Gensicke, Daniel Staub, Thomas Wolff, Edin Mujagic, Ioannis Tsogkas, Marios Psychogios, M Eline Kooi, Stefan T Engelter, Philippe Lyrer, Leo Bonati, on behalf of the BIOPLAQUE Collaborative Group

**Affiliations:** 1 Department of Neurology and Stroke Center, University Hospital Basel and University of Basel, Basel, Switzerland; 2 Cardiovascular Research Institute Maastricht (CARIM), Maastricht University, Maastricht, the Netherlands; 3 Department of Radiology and Nuclear Medicine, Maastricht University Medical Center+ (MUMC+), Maastricht, the Netherlands; 4 Clinical Trial Unit, University Hospital Basel and University of Basel, Basel, Switzerland; 5 Department of Neurosurgery, Inselspital, Bern University Hospital, University of Bern, Bern, Switzerland; 6 Department of Rehabilitation and Neurology, University Department of Geriatric Medicine FELIX PLATTER, University of Basel, Basel, Switzerland; 7 Vascular Medicine/Angiology, University Hospital Basel and University of Basel, Basel, Switzerland; 8 Department of Vascular Surgery, University Hospital Basel and University of Basel, Basel, Switzerland; 9 Department of Diagnostic and Interventional Neuroradiology, University Hospital Basel and University of Basel, Basel, Switzerland; 10 Research Department, Reha Rheinfelden, Rheinfelden, Switzerland; 11 Department of Neurology and Stroke Center, University Hospital Basel and University of Basel, Basel, Switzerland; 12 Research Department, Reha Rheinfelden, Rheinfelden, Switzerland; 13 Department of Neurology and Stroke Center, University Teaching and Research Hospital, Health Eastern Switzerland (HOCH), Cantonal Hospital St. Gallen, St. Gallen, Switzerland; 14 Department of Rehabilitation and Neurology, University Department of Geriatric Medicine FELIX PLATTER, University of Basel, Basel, Switzerland; 15 Clinical Trial Unit, University Hospital Basel and University of Basel, Basel, Switzerland; 16 Department of Diagnostic and Interventional Neuroradiology, University Hospital Basel and University of Basel, Basel, Switzerland; 17 Vascular Medicine/Angiology, University Hospital Basel and University of Basel, Basel, Switzerland; 18 Department of Vascular Surgery, University Hospital Basel and University of Basel, Basel, Switzerland; 19 Department of Neurosurgery, Inselspital, Bern University Hospital, University of Bern, Bern, Switzerland; 20 Cardiovascular Research Institute Maastricht (CARIM), Maastricht University, Maastricht, The Netherlands; 21 Department of Radiology and Nuclear Medicine, Maastricht University Medical Center+ (MUMC), Maastricht, The Netherlands; 22 Stroke Center, Klinik Hirslanden Zurich, Zurich, Switzerland

**Keywords:** carotid artery disease, carotid plaque, duplex ultrasound, intraplaque hemorrhage, lipid-rich necrotic core, magnetic resonance angiography (MRA)

## Abstract

**Background:**

Intraplaque hemorrhage (IPH) and lipid-rich necrotic core (LRNC) are key markers of carotid plaque vulnerability and stroke risk. Though magnetic resonance imaging (MRI) can detect both, duplex ultrasound is more accessible and may identify echolucent plaque areas that correlate with IPH or LRNC. This study investigated whether quantitative ultrasound can predict the presence of IPH or LRNC in atherosclerotic carotid artery stenosis (CS).

**Methods:**

In this prospective single-center study, patients with moderate to severe asymptomatic or symptomatic CS underwent MR plaque imaging and quantitative ultrasound with color mapping. Echolucency was measured in various plaque areas using several gray-scale thresholds. IPH was defined as part of the LRNC. Receiver operating characteristic (ROC) curve analysis assessed the predictive value of ultrasound for MRI-detected IPH or LRNC.

**Results:**

Among 113 enrolled patients, 75 patients (mean age 75 years; 69% men; 40% with symptomatic CS) were included in the analysis. On MRI, 43 patients (57%) had LRNC, and 32 patients (43%) showed IPH in the index artery. In the group without IPH, LRNC status could not be scored for 19 index arteries. Echolucency of the plaque surface with a gray-scale value < 20 showed the strongest association with IPH, with an area under the ROC curve (AUC) of 0.58 (95% CI 0.43, 0.71) and a negative predictive value of 0.64 (95% CI 0.50, 0.69) for the presence of IPH (sensitivity 0.50, specificity 0.65). For LRNC without IPH, several thresholds yielded the best-performing AUC of 0.48 (95% CI 0.23, 0.73/0.74)

**Conclusion:**

Quantitative ultrasound does not reliably predict the presence of IPH or LRNC, as detected by MRI, in patients with atherosclerotic internal CS.

## Background

Approximately 15–20% of ischemic strokes are caused by atherosclerotic stenosis of the internal carotid artery (ICA).^1,2^ The degree of stenosis and associated symptoms have long served as the primary criteria for risk stratification and guiding treatment decisions.^3^ A growing body of evidence supports the concept of a ‘vulnerable plaque’ prone to rupture and causing stroke independent of the degree of stenosis.^4^

Vulnerable carotid plaques are typically characterized by intraplaque hemorrhage (IPH), a large lipid-rich necrotic core (LRNC) and a thin fibrous cap.^5–8^ Previous observational studies and meta-analyses have reported an increased risk for future ipsilateral ischemic events (including stroke, transient ischemic attack [TIA], and amaurosis fugax) in the presence of LRNC or IPH.^9–13^ IPH was associated with a four- to 12-fold increased risk of ipsilateral cerebral ischemic events, rendering it a substantially stronger predictor of stroke risk than the degree of stenosis or symptom status.^9,10^

Magnetic resonance imaging (MRI) is widely recognized as the imaging modality of choice for in vivo detection of IPH and LRNC,^12,14^ and is increasingly used to identify patients at risk of stroke who may benefit from carotid revascularization; it was therefore chosen as the gold standard in our study. Histological measurement was not feasible, as only a few patients underwent carotid endarterectomy. The magnetization-prepared rapid acquisition gradient echo (MP-RAGE) sequence is well validated for visualizing IPH, demonstrating high sensitivity and specificity (80% and 97%, respectively), with histological measurements as the reference standard, and high interobserver agreement (κ = 0.73, 95% CI 0.53, 0.92).^15,16^ Thus, MP-RAGE is commonly recommended for the detection of IPH. Alternatively, precontrast (‘mask’) images of contrast-enhanced magnetic resonance angiography (CE-MRA) can be used to score the presence of IPH in case MP-RAGE images are not available. Interobserver agreements for identifying IPH on MP-RAGE and mask images were excellent (κ = 0.93 and 0.96, respectively).^14^ LRNC presents as a focal hypointense area on T2-weighted images and is best appreciated on postcontrast dark blood T1-weighted turbo spin echo (TSE) images as a focal nonenhancing area.^12,14,17^

Ultrasound, which is often more easily accessible than MRI, is routinely used to classify the degree of ICA stenosis. In addition, high-resolution B-mode ultrasound allows characterization of the arterial wall, including the size and echogenicity of the atherosclerotic plaque.^18^ Plaques appearing echolucent (dark) on B-mode ultrasound are characterized by higher lipid content, less calcification, and less fibrous tissue in histological examination.^19^ Several classifications of plaque echogenicity exist and plaque echolucency was shown to be associated with an elevated risk of stroke and coronary events.^10,20,21^ However, an important limitation of ultrasound is that it cannot reliably distinguish between lipid and hemorrhage within the plaque based on echogenicity.^22^ Visual plaque analysis is limited by poor inter- and intraobserver agreement.^23^ This limitation led to computer-assisted, operator-independent measurement methods for evaluating the gray-scale median (GSM) of plaque echogenicity.^24^ The GSM value is a measure of overall plaque echogenicity and is determined by the median gray-scale value within the segmented plaque normalized to the lumen and echogenic adventitia layer.^25^ Several studies have found that GSM may be a valuable parameter for predicting the risk of cerebrovascular events;^18^ however, other studies have not found such an association.^26^ A method using regional plaque analysis with color mapping showed that echogenicity at the plaque surface differs between patients with symptomatic and asymptomatic carotid stenosis, particularly in combination with the degree of stenosis.^25,27^

The aim of this exploratory study was to test the hypothesis that quantitative gray-scale measurement on B-mode ultrasound predicts the presence of IPH or LRNC detected by MRI in atherosclerotic internal carotid artery stenosis.

## Methods

### Study population

This study is a single-center, prospective, observational study entitled BIOPLAQUE, which was conducted at the University Hospital of Basel, Switzerland, between February 2012 and October 2020. BIOPLAQUE was approved by the responsible local ethics review board and was designed as a sub-study nested in large randomized controlled trials. Patients eligible for the BIOPLAQUE study met the inclusion criteria for either the Second Asymptomatic Carotid Surgery Trial (ACST-2) or the Second European Carotid Surgery Trial (ECST-2).^28,29^

The detailed methods of ACST-2 and ECST-2 have been published elsewhere.^28,29^ Briefly, ACST-2 randomized patients with a moderate to severe asymptomatic carotid artery stenosis, in whom carotid intervention was considered necessary, to either carotid endarterectomy (CEA) or carotid stenting (CAS).^28^ Patients in ECST-2 had at least a 50% symptomatic carotid artery stenosis (defined by the occurrence of ipsilateral ischemic symptoms in the past 6 months [retinal infarction, amaurosis fugax, TIA, or ischemic stroke]), where the treating clinician determined whether the symptoms were related to the stenosis, or asymptomatic carotid stenosis with an estimated moderate 5-year risk of stroke of < 20%, calculated by the Carotid Artery Risk (CAR) score. Patients were randomized to either optimized medical treatment (OMT) alone or OMT combined with revascularization by CEA or CAS.^29^ Exclusion criteria were: (i) prior CEA or CAS on the side of the index carotid artery; (ii) fresh thrombus on the side of the index carotid artery; (iii) prior neck irradiation; (iv) pregnancy; and (v) contraindication for MRI (i.e., cardiac pacemaker, metal implant, claustrophobia precluding MRI). Carotid duplex ultrasound was mandatory in both studies to determine the degree of stenosis, and had to be confirmed by angiography (usually computed tomography [CT]- or MR-based angiography).^28,29^

Weekly interdisciplinary meetings involving neurologists, neuroradiologists, and vascular surgeons are held to evaluate patient eligibility for stenting or endarterectomy. Patients eligible for ACST-2 or ECST-2 were additionally asked to participate in the BIOPLAQUE study. Written informed consent was obtained from all participants.

Patients were examined clinically at baseline by a neurologist. Focal neurological deficits were measured using the National Institutes of Health Stroke Scale, and the functional level of independence using the modified Rankin Scale.^30,31^ In the current analysis, we included only patients who underwent a baseline carotid MRI examination 1–7 days before treatment, if randomized to an invasive treatment, or promptly after signed consent if treated with best medical treatment only.

### Magnetic resonance imaging (MRI) acquisition

The preplanned MRI protocol included diffusion-weighted imaging (DWI) to detect acute ischemic brain lesions, susceptibility-weighted imaging (SWI) to measure cerebral microbleed burden, and fluid-attenuated inversion recovery (FLAIR) sequences to measure cerebral white matter changes and chronic infarctions. As previously described,^14^ carotid plaque MRI included MP-RAGE, CE-MRA (with mask images available), time-of-flight (TOF), T1-weighted TSE, T1-weighted BLADE, and T2-weighted sequences. For the CE-MRA series, a 3D fast field echo sequence was acquired before (i.e., the mask images) and after intravenous injection of 0.1 mmol/kg body weight of gadobutrol (Gadavist, Bayer Schering, Leverkusen, Germany). MRI field strengths were 1.5-tesla or 3-tesla (Siemens Healthineers, Erlangen, Germany). The parameters of the carotid MR sequences are presented in Table 1.

**Table 1.** Magnetic resonance imaging parameters of the carotid plaque examination.

Pulse sequence	MP-RAGE	CE-MRA	T1-weighted BLADE
Vendor	Siemens	Siemens	Siemens
Scanner type	Prisma	Prisma	Prisma
Acquisition format	3D	3D	2D
Acquisition plane	transversal	coronal	transversal
Sequence name	tfl3d1_16	fl3d1	tirB2d1_9
TR (ms)	884	3.0	1500
TE (ms)	5.27	1.1	53
TI (ms)	500	NA	659
Flip angle (°)	15	22	60
No. of slices	128	88	18
Slice thickness (mm)	0.6	0.9	2.0
FOV (mm)	160 × 160	297 × 340	160 × 160
Acquisition matrix	256 × 256	210 × 320	320 × 320
Acquired voxel size (mm)	0.63 × 0.63 × 1.26	1.42 × 1.06 × 147	0.5 × 0.5 × 2
Reconstruction matrix	256 × 256	512 × 512	320 × 320
Reconstructed voxel size (mm)	0.6 × 0.6 × 0.6	0.6 × 0.7	0.5 × 0.5
Echo train length	1	0	9
Parallel imaging	No	Yes	Yes
No. of signal averages	1	1	1
Fat suppression	Yes	No	No

CE-MRA, contrast-enhanced magnetic resonance angiography; FOV, field of view; MP-RAGE, magnetization-prepared rapid acquisition gradient; NA, not applicable; TE, echo time; TI, inversion time; TR, repetition time.

### MR image review and criteria

MR plaque images were reviewed at the University of Maastricht using dedicated vessel wall image analysis software (VesselMass; Leiden University Medical Centre, the Netherlands). Transversal reconstructed images of MP-RAGE and mask sequences were anonymized and one trained observer (MK) qualitatively scored the presence of IPH and LRNC in both internal carotid arteries simultaneously, blinded to the carotid index side, symptom status, duplex ultrasound findings, and clinical outcome.

**Figure 1.**
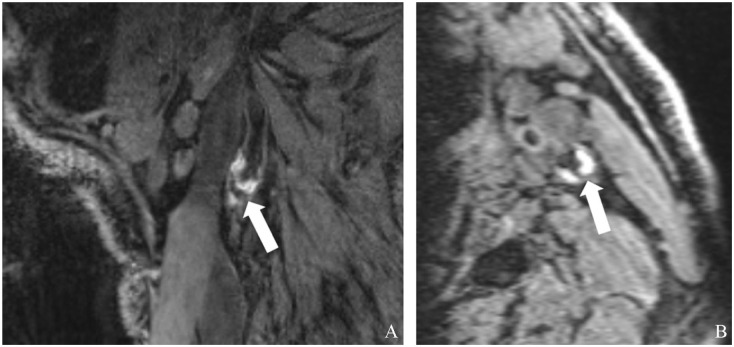
Representative magnetization-prepared rapid acquisition gradient (MP-RAGE) magnetic resonance sequence showing intraplaque hemorrhage in a carotid plaque (white arrows): **(A)** sagittal view and **(B)** axial view.

The presence of IPH was defined as a hyperintense signal within the bulk of the plaque compared to the adjacent sternocleidomastoid muscle and was scored as published previously.^14^ IPH was scored on MP-RAGE images and, if not available, on mask images of CE-MRA. A four-point certainty score was assigned for scoring the presence of IPH (4: very certain; 1: uncertain). Cases with a low certainty score (< 2) were excluded from the analysis. Figure 1 shows representative MR images of a plaque with IPH.

LRNC was identified on pre- and postcontrast T1-weighted TSE images, or, if not available, on T1-weighted BLADE images. When neither TSE nor BLADE sequences were available, T2-weighted images were used to score LRNC. A five-point image quality score was applied (5: excellent; 1: poor) based on the overall signal-to-noise ratio and clarity of the vessel wall. Cases with a low-quality score (⩽ 2) were excluded from the analysis.^14,32^ Of note, IPH was considered part of an LRNC. Thus, all patients with an IPH have an LRNC by definition, whereas not all patients with an LRNC also have an IPH.

### Ultrasound imaging

Carotid duplex ultrasound was performed 1–7 days before treatment if randomized to an invasive treatment, or promptly after signed consent if treated with best medical treatment only, by experienced sonographers to determine the degree of internal carotid stenosis and to obtain B-mode images of the carotid plaque. The degree of stenosis was measured by duplex ultrasound based on flow velocities and categorized in 10% bands using criteria which equate to the degree of stenosis measured by angiography according to the method used in the NASCET (North American Symptomatic Carotid Endarterectomy Trial).^33^ Plaque morphology was evaluated ‘online’ from the B-mode images, as previously described.^34^ All investigations were performed using ultrasound devices (Philips iU22 [Philips AG, Switzerland] and Siemens [Siemens AG, Switzerland]) with a 7-MHz transducer.

**Figure 2.**
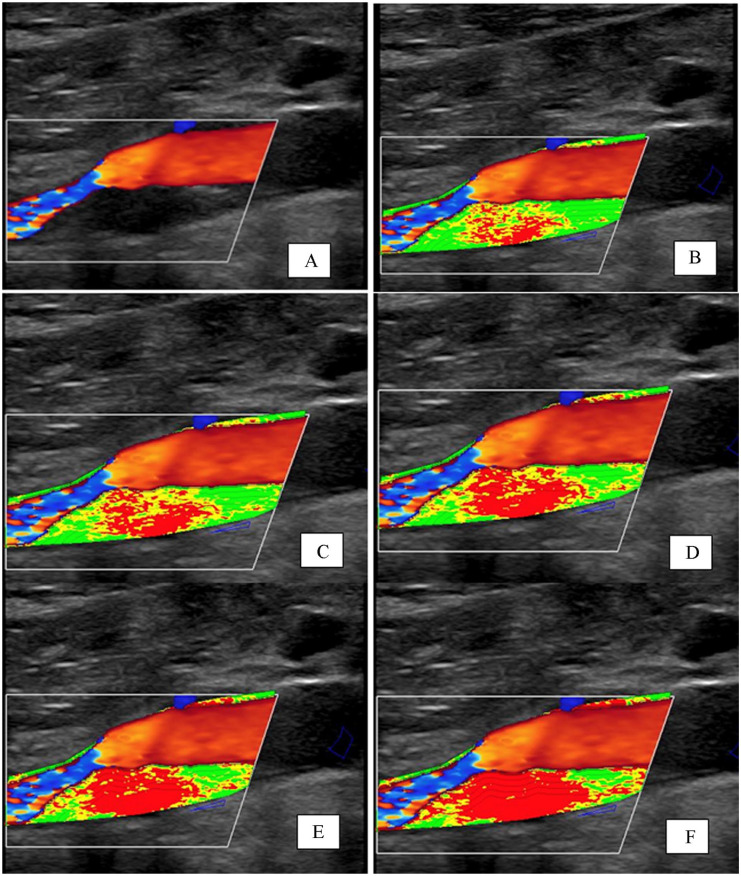
Representative B-mode image showing a hypoechoic carotid plaque **(A)** and the corresponding color mapping with the following thresholds: **(B)** lowest gray-scale values (< 20 mapped in red), intermediate values (20–50 mapped in yellow), and highest values (> 50 mapped in green); **(C)** < 30 mapped in red, 30–60 mapped in yellow, > 60 mapped in green; **(D)** < 40 mapped in red, 40–70 mapped in yellow, > 70 mapped in green; **(E)** < 50 mapped in red, 50–80 mapped in yellow, > 80 mapped in green; and **(F)** < 60 mapped in red, 60–90 mapped in yellow, > 90 mapped in green.

Quantitative gray-scale analysis of B-mode images was performed offline with the use of a computer-aided analysis program (Plaque Analyzer; HeartFlow, Inc.), which gives a layer-by-layer analysis of plaque echolucency. The GSM of the whole plaque was assessed according to the method described by El-Barghouty et al.^24^ The gray-scale values of the plaque were normalized by automatic linear scaling after outlining a black region in the perfused vessel lumen (blood with a gray-scale = 0) and a bright region in the vessel adventitia (gray-scale value = 190) to provide reproducible gray-level measurements independent of ambient light, gain adjustments, and different scanners. Each pixel of the carotid plaque was then mapped in three different colors according to the intensity of echogenicity and its corresponding gray-scale value: red (echolucent), yellow (intermediate), and green (echogenic). Five different thresholds were considered for echolucency (red): gray-scale values of 60, 50, 40, 30, and 20. For each plaque, the proportion of each color present in the whole plaque and at its surface was assessed automatically.^25^ Figure 2 shows a representative image of an echolucent plaque, along with the corresponding color mapping based on various gray-scale values.

For this analysis, we defined the plaque surface as the outer one-third of the total plaque thickness facing the lumen. Using five different gray-scale threshold values, both in the entire plaque and in the plaque surface, we obtained 10 quantitative measures for the percentage of the echolucent area. In addition, the GSM of the whole plaque was calculated. Patients, in whom acoustic shadowing due to calcifications made it impossible to visualize the plaque, or those with calcifications involving more than 50% of the plaque length, were excluded.

All sonographic analyses were performed at the University Hospital of Basel. The Plaque Analyzer reading was performed by two trained observers (BW and SM), who analyzed the images by consensus while blinded to clinical symptoms and MRI findings. In two cases of disagreement about the quality of the color mapping, a third, experienced neurologist (LB) made the decision to include or exclude the patients.

### Statistical analysis

All calculations were made with SAS (version 9.4; SAS Institute, Inc.) and R (version 4.3; R Foundation for Statistical Computing). Categorical and continuous variables are shown respectively as numbers and percentages (%) or as means with SD.

The primary aim was to determine, among the 11 predefined quantitative ultrasound measures of plaque echolucency, the one that best predicted the presence of IPH on MRI. A particular challenge for detecting IPH on ultrasound is plaques with an LRNC, as fat and blood cannot reliably be distinguished on ultrasound images. However, we hypothesized that they appear at different depths of the plaque. Therefore, we investigated ultrasound measurements from the surface and from the whole plaque separately. As a secondary aim, we investigated which ultrasound measurement best distinguishes plaques with an LRNC from plaques without an LRNC, when considering only patients without IPH.

We performed receiver operating characteristic (ROC) curves and calculated the associated area under the ROC curves (AUC) for each of the 11 measures. We report estimated AUCs and bootstrap-based 95% CIs. Statistical significance was defined as *p* < 0.05. In addition, the negative predictive values (NPVs) as well as the sensitivities and specificities for the best six measures were determined.

## Results

**Figure 3.**
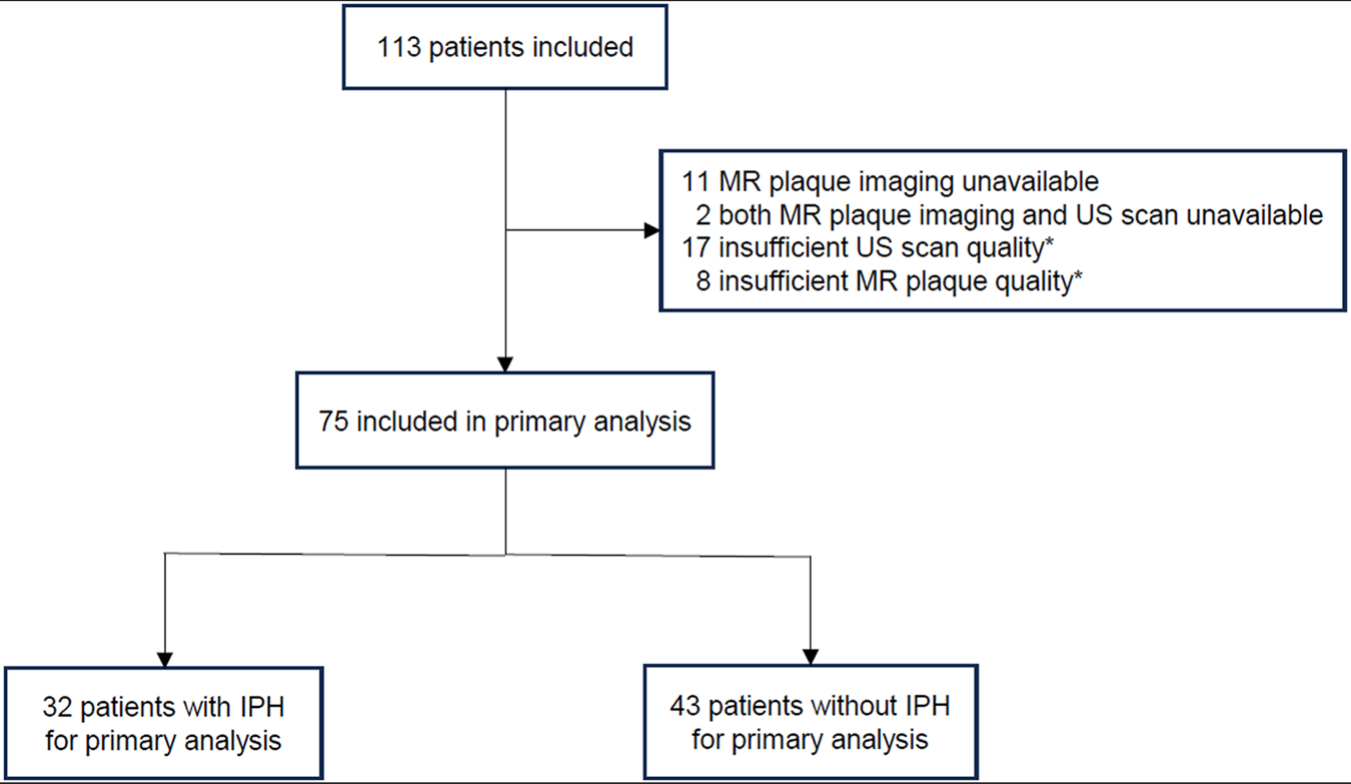
Study profile for the primary analysis assessing intraplaque hemorrhage. *Of the 17 ultrasound scans, four had insufficient image quality, and 13 showed too much calcification (> 50% of the plaque) to allow adequate gray-scale measurement. Additionally, eight patients were excluded because of insufficient MR plaque imaging: four had a low certainty score (< 2) for IPH assessment, and four had only time-of-flight sequences available. IPH, intraplaque hemorrhage; MR, magnetic resonance; US, ultrasound.

A total of 113 consecutive patients were enrolled in this prospective observational study. A total of 38 patients were excluded because of missing MR plaque imaging (*n* = 11), missing both MR and ultrasound scans (*n* = 2), insufficient MR plaque image quality (*n* = 8), or insufficient ultrasound image quality (*n* = 17) (Figure 3). Ultimately, 75 patients were included in the primary analysis (mean age 75 years, 31% women). In 32 patients (43%), an IPH was present in the index artery (Table 2). All index carotid arteries (100%) with an IPH also had an LRNC, as IPH was considered to be part of the LRNC, in line with previous studies.^26^ LRNC was also detected on MRI in 11 (46%) index arteries without IPH (the LRNC status could not be scored due to low image quality or available sequences for 19 index arteries) (Table 3).

**Table 2.** Demographics and baseline characteristics in patients with intraplaque hemorrhage (IPH+) versus without intraplaque hemorrhage (IPH–) in the index artery.

	IPH+ (*n* = 32)	IPH– (*n* = 43)
Male sex	21 (65.6)	31 (72.1)
Age in years, mean [range]	76 [57–87]	74 [48–88]
Carotid artery, left	18 (56.3)	15 (34.9)
Ipsilateral carotid stenosis^a^		
50–69%	14 (43.8)	6 (14.0)
70–79%	12 (37.5)	19 (44.2)
80–99%	6 (18.8)	18 (41.9)
Contralateral carotid stenosis^a^		
< 50%	22 (68.8)	26 (60.5)
50–69%	5 (15.6)	7 (16.3)
70–99%	1 (3.1)	4 (9.3)
Occlusion	4 (12.5)	6 (14.0)
Index event		
Asymptomatic	15 (46.9)	32 (74.4)
Symptomatic		
Amaurosis fugax	2 (6.3)	6 (14.0)
TIA	5 (15.6)	1 (2.3)
Ischemic stroke	10 (31.3)	4 (9.3)
Previous TIA or stroke^b^		
Ipsilateral (> 6 months ago)	5 (15.6)	5 (11.6)
Contralateral	7 (21.9)	14 (32.6)
Vertebrobasilar	4 (12.5)	5 (11.6)
Medical history		
Dyslipidemia	32 (100.0)	40 (93.0)
Hypertension	27 (84.4)	35 (81.4)
Coronary heart disease	11 (34.4)	13 (30.2)
Diabetes mellitus	8 (25.0)	10 (23.3)
Renal impairment	5 (15.6)	7 (16.3)
Peripheral artery disease	5 (15.6)	5 (11.6)
Atrial fibrillation	3 (9.4)	6 (14.0)
BMI [SD]	26.4 [3.7]	26.2 [3.9]
Smoking^c^		
Current	6 (19.4)	14 (32.6)
Former	14 (45.2)	16 (37.2)

Values are presented as *n* (%) unless otherwise noted.

a According to the NASCET-based criteria.

b TIA included amaurosis fugax; stroke included retinal infarction.

c Smoking status unknown: one patient in IPH+.

BMI, body mass index; IPH, intraplaque hemorrhage; NASCET, North American Symptomatic Carotid Endarterectomy Trial; TIA, transient ischemic attack.

**Table 3.** Prevalence of intraplaque hemorrhage and lipid-rich necrotic core in symptomatic compared to asymptomatic index arteries.

	Symptomatic carotid (*n* = 28)	Asymptomatic carotid (*n* = 47)
Presence of IPH, *n* (%)	17 (60.7)	15 (31.9)
Presence of LRNC, *n* (%)	22^a^ (88.0)	21^a^ (67.7)

a LRNC status unknown in three symptomatic and 16 asymptomatic carotid arteries (included in primary IPH analysis).

IPH, intraplaque hemorrhage; LRNC, lipid-rich necrotic core.

On the contralateral carotid artery, IPH status could not be scored in 17 arteries; two of these were occluded. Of the remaining 62 patients, IPH was present in 10 (16%) patients, whereas 52 (84%) had no IPH. A total of five patients had IPH on both carotid arteries. All contralateral arteries with IPH had, by definition, an LRNC (100%). Among the contralateral arteries without IPH, the LRNC status could not be scored in 16 because of low image quality or unavailable sequences; of the remaining arteries, 12 (33%) had an LRNC and 24 (67%) did not.

Thirty patients (40% of the study population) had symptomatic carotid stenosis. Among these, the index event was an ipsilateral ischemic stroke in 15 cases (50%), amaurosis fugax in nine (30%), and TIA in six (20%) – each, by definition, attributable to the carotid stenosis. Compared to the group without IPH, the group with IPH were less often men (IPH 66% vs no-IPH 72%), less often had a high-grade (70–99%) stenosis (IPH 56% vs no-IPH 86%), and more often a left-sided index stenosis (IPH 56% vs no-IPH 35%) (Table 2). IPH was present in 61% (17/28) of symptomatic index carotid stenoses (Table 3).

Plaque surface echogenicity, using a cut-off gray-scale value of < 20, showed the best prediction of IPH, with an AUC of 0.58 (95% CI 0.43, 0.71) (Table 4). The negative predictive value at the optimal cut-off value of 47.7%, using a threshold of < 20 for plaque surface echolucency (color mapping in red), was 0.64 (CI 0.50, 0.69) for detecting IPH. This threshold demonstrated a sensitivity of 0.50 and a specificity of 0.65 (Supplemental Figure 1). In contrast, the best prediction for LRNC without IPH was observed for several thresholds: GSM, whole-plaque echogenicity using a gray-scale cut-off value of < 50, and plaque surface echogenicity using gray-scale cut-off values of < 50 or < 60 (AUCs 0.48, 95% CI 0.23, 0.73 to 0.74; Supplemental Table 1). The negative predictive cut-off value for detecting LRNC without IPH using GSM or a threshold of < 50 for whole-plaque echolucency was 0.65 (CI 0.01, 0.68) and 0.65 (CI 0.14, 0.68), respectively. Both thresholds had a sensitivity of 0.45 and a specificity of 0.85.

**Table 4.** Quantitative ultrasound measurements for the prediction of intraplaque hemorrhage.

Plaque echogenicity thresholds	AUC	95% CI
Percentage of low echogenicity under the surface (red < 20)	0.58	[0.43, 0.71]
Percentage of low echogenicity under the surface (red < 40)	0.56	[0.42, 0.68]
Percentage of low echogenicity under the surface (red < 30)	0.55	[0.42, 0.69]
Percentage of low echogenicity under the surface (red < 50)	0.53	[0.40, 0.66]
Percentage of low echogenicity of the whole plaque (red < 20)	0.52	[0.38, 0.66]
Percentage of low echogenicity under the surface (red < 60)	0.52	[0.39, 0.66]
Percentage of low echogenicity of the whole plaque (red < 30)	0.50	[0.37, 0.64]
Percentage of low echogenicity of the whole plaque (red < 40)	0.49	[0.35, 0.63]
Gray-scale median (whole plaque)	0.47	[0.34, 0.60]
Percentage of low echogenicity of the whole plaque (red < 50)	0.46	[0.33, 0.60]
Percentage of low echogenicity of the whole plaque (red < 60)	0.45	[0.32, 0.58]

AUC, area under the curve.

## Discussion

This prospective explorative study showed that quantitative gray-scale analysis on B-mode ultrasound is not able to reliably predict the presence of IPH on MRI. We used computer-assisted gray-scale analysis to quantitatively assess plaque echogenicity in B-mode ultrasound images.^24^ Our hypothesis was that certain patterns of echogenicity would predict the presence or absence of IPH on MRI. Previous research showed that plaques with hemorrhage and increased lipid content are associated with lower gray-scale median values, whereas those with higher fibrous tissue content tend to display higher gray-scale values.^35,36^ Identifying such patterns on ultrasound might allow tailoring of the indication for plaque MRI, which is still less accessible than ultrasound in routine clinical practice. Research comparing findings on ultrasound and plaque MRI is scarce and has yielded inconsistent results.^12^

In our study, we observed that the proportion of the echolucent plaque area defined by a gray-scale value < 20 close to the plaque surface showed a tendency toward discrimination between plaques with and without IPH. Considering the confidence interval, the observed effect may, however, be due to chance. We did not find a strong correlation between IPH and GSM, which may be explained by the fact that GSM measures the overall echodensity of the plaque without accounting for the spatial distribution of echoes within the plaque.^25,36^ The association between low gray-scale values under the plaque surface and IPH is consistent with previous research.^25,37^ In contrast, plaques with an LRNC but no IPH tended to be less echolucent than plaques with IPH. However, the predictive performance was no better than random.

Previous studies have demonstrated that LRNC is significantly larger in plaques containing IPH than in those without.^38^ A longitudinal study of asymptomatic low-grade carotid stenosis showed that IPH is associated with plaque progression.^39^ IPH may drive the stenotic phenotype. Moreover, the amount of IPH positively correlates with lipid content and inversely with fibrous tissue within the plaque.^19^ However, distinguishing between IPH and LRNC remains challenging, even on histopathological examination, and in many instances, it is difficult to reliably differentiate the two.^17^

It is well established that there is a close relationship between the amount of hemorrhage and lipid content within the plaque, which was first demonstrated nearly 30 years ago.^19^ This relationship led to the term ‘soft tissue’ to describe these combined features.^32^ As IPH typically occurs within the LRNC, we consider IPH as part of the LRNC, in line with other studies. Consequently, plaques with IPH are, by definition, also classified as having an LRNC. On the other hand, plaques may have an LRNC but no IPH.^26^

IPH was present in MR plaque imaging in nearly twice as many symptomatic plaques as asymptomatic plaques (61% vs 32%), and LRNC was more commonly observed in symptomatic plaques (88% vs 68%). These findings support the existing literature suggesting that both IPH and LRNC are more prevalent in symptomatic carotid plaques, even when the degree of stenosis is similar.^9,10,13,22^

Although several studies have shown that LRNC is located closer to the lumen in symptomatic plaques compared to asymptomatic plaques, none have specified whether this applies to plaques with or without IPH.^5,25,37,40^ This raises the question of why plaques with both IPH and LRNC exhibit particularly low echogenicity under the plaque surface – although this observation did not reach statistical significance.

In ruptured plaques, the LRNC typically occupies one-third to one-half of the total plaque area, whereas in unruptured plaques it generally comprises less than one-quarter.^6^ However, ruptured plaques are often associated with IPH as well.

## Limitations

The study has several limitations. First, our results are limited by a relatively small sample size. This study did not include a formal a priori power calculation because the sample size was defined by the number of consecutive patients meeting the inclusion criteria, and no robust data on expected diagnostic effect sizes were available. As a result, the analysis was designed as a prospective, exploratory, and hypothesis-generating study, providing estimates for future, adequately powered investigations.

Second, in our setting, gray-scale ultrasound measured echogenicity on a continuous scale, which may not provide the resolution required to detect subtle differences in plaque composition. This limits the ability to identify fine variations in plaque structure.

Third, we used MR findings for comparison, though histology of excised carotid specimens is still considered the gold standard for evaluating plaque features. The latter was not possible because such specimens were not available in the setting of our study, not least because not all patients underwent carotid endarterectomy. However, the MRI technique used to detect IPH is well-established and known for its high sensitivity and specificity, making it the most effective in vivo modality for IPH detection. Furthermore, no prior study or classification has specifically addressed the different stages of IPH.^41^ As IPH evolves over time, both its magnetic resonance properties and histological characteristics undergo changes, with the organization gradually replacing fibrin with a more collagen-like extracellular matrix structure.^32,42^ The Derksen classification, the most comprehensive and widely used in anatomopathology, distinguishes four stages of IPH^43^; however, no currently existing imaging technique can accurately discriminate between these stages.^41^

Finally, we did not fully characterize all features of vulnerable plaque. For instance, ulceration is best detected using CT angiography, which was not available for all patients. Additionally, fibrous cap status could not be reliably assessed in all cases and was therefore excluded from the analysis. These limitations may have influenced the results of our gray-scale analysis.

## Conclusion

Quantitative ultrasound did not reliably predict the presence or absence of IPH or LRNC, as detected by MRI, in patients with atherosclerotic internal carotid artery stenosis. Therefore, if MR plaque imaging is being considered to guide risk assessment or treatment decisions, it should be performed irrespective of ultrasound findings.

## Supplemental Material

sj-docx-1-vmj-10.1177_1358863X251410527 – Supplemental material for Diagnostic accuracy of gray-scale analysis on B-mode ultrasound for identifying intraplaque hemorrhage and lipid-rich necrotic core in carotid plaques Supplemental material, sj-docx-1-vmj-10.1177_1358863X251410527 for Diagnostic accuracy of gray-scale analysis on B-mode ultrasound for identifying intraplaque hemorrhage and lipid-rich necrotic core in carotid plaques by Benjamin Wagner, Sasha Mukhija, Mohamed Kassem, Andrea Wiencierz, Mandy D Müller, Henrik Gensicke, Daniel Staub, Thomas Wolff, Edin Mujagic, Ioannis Tsogkas, Marios Psychogios, M Eline Kooi, Stefan T Engelter, Philippe Lyrer and Leo Bonati in Vascular Medicine

sj-docx-2-vmj-10.1177_1358863X251410527 – Supplemental material for Diagnostic accuracy of gray-scale analysis on B-mode ultrasound for identifying intraplaque hemorrhage and lipid-rich necrotic core in carotid plaques Supplemental material, sj-docx-2-vmj-10.1177_1358863X251410527 for Diagnostic accuracy of gray-scale analysis on B-mode ultrasound for identifying intraplaque hemorrhage and lipid-rich necrotic core in carotid plaques by Benjamin Wagner, Sasha Mukhija, Mohamed Kassem, Andrea Wiencierz, Mandy D Müller, Henrik Gensicke, Daniel Staub, Thomas Wolff, Edin Mujagic, Ioannis Tsogkas, Marios Psychogios, M Eline Kooi, Stefan T Engelter, Philippe Lyrer and Leo Bonati in Vascular Medicine

sj-docx-3-vmj-10.1177_1358863X251410527 – Supplemental material for Diagnostic accuracy of gray-scale analysis on B-mode ultrasound for identifying intraplaque hemorrhage and lipid-rich necrotic core in carotid plaques Supplemental material, sj-docx-3-vmj-10.1177_1358863X251410527 for Diagnostic accuracy of gray-scale analysis on B-mode ultrasound for identifying intraplaque hemorrhage and lipid-rich necrotic core in carotid plaques by Benjamin Wagner, Sasha Mukhija, Mohamed Kassem, Andrea Wiencierz, Mandy D Müller, Henrik Gensicke, Daniel Staub, Thomas Wolff, Edin Mujagic, Ioannis Tsogkas, Marios Psychogios, M Eline Kooi, Stefan T Engelter, Philippe Lyrer and Leo Bonati in Vascular Medicine
