# Supplementary material for: Diagnostic accuracy of gray-scale analysis on B-mode ultrasound for identifying intraplaque hemorrhage and lipid-rich necrotic core in carotid plaques
Source: Vasc Med. 2026 Feb 25;31(2):175–84. doi: 10.1177/1358863X251410527 (PMC13109598; doi:10.1177/1358863X251410527)
Supplement: sj-docx-1-vmj-10.1177_1358863X251410527 – Supplemental material for Diagnostic accuracy of gray-scale analysis on B-mode ultrasound for identifying intraplaque hemorrhage and lipid-rich necrotic core in carotid plaques [file sj-docx-1-vmj-10.1177_1358863X251410527.docx]

**
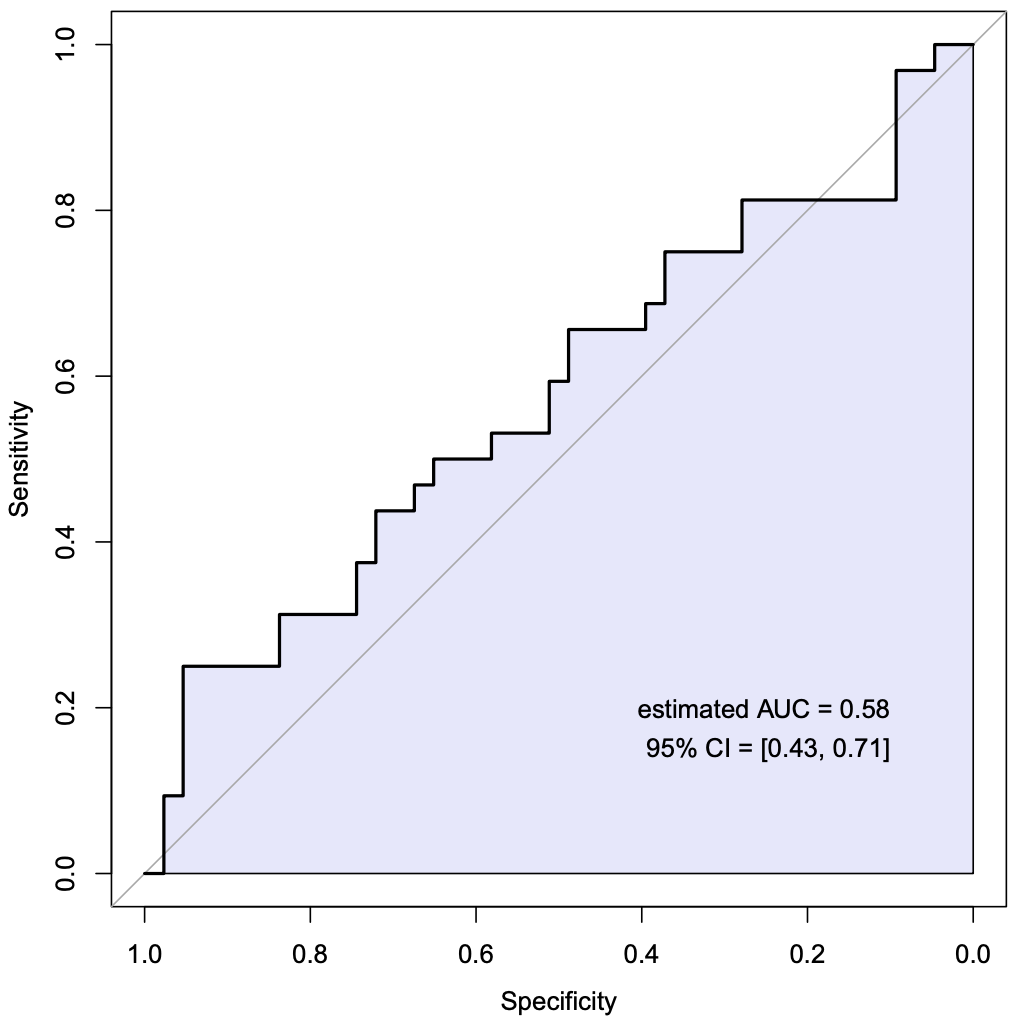
**

ROC plaque surface low echogenicity <20 for predicting intraplaque haemorrhage

**Supplemental figure 1:** Receiver operating characteristic (ROC) curve analysis for determining the most appropriate grey-scale cut-off value to classify presence or absence of plaque haemorrhage as defined by MR plaque imaging

AUC, area under the curve; CI, confidence interval
