# Supplementary material for: Diagnostic accuracy of gray-scale analysis on B-mode ultrasound for identifying intraplaque hemorrhage and lipid-rich necrotic core in carotid plaques
Source: Vasc Med. 2026 Feb 25;31(2):175–84. doi: 10.1177/1358863X251410527 (PMC13109598; doi:10.1177/1358863X251410527)
Supplement: sj-docx-2-vmj-10.1177_1358863X251410527 – Supplemental material for Diagnostic accuracy of gray-scale analysis on B-mode ultrasound for identifying intraplaque hemorrhage and lipid-rich necrotic core in carotid plaques [file sj-docx-2-vmj-10.1177_1358863X251410527.docx]

**Supplemental material**

**Supplemental Table 1: Quantitative ultrasound measurements for prediction of LRNC**

| Plaque echogenicity thresholds | AUC | 95% CI |
| --- | --- | --- |
| Grey-scale median (whole plaque)  Percentage of low echogenicity under the surface (red < 60)  Percentage of low echogenicity of the whole plaque (red < 50)  Percentage of low echogenicity under the surface (red < 50)  Percentage of low echogenicity of the whole plaque (red < 60)  Percentage of low echogenicity under the surface (red < 40)  Percentage of low echogenicity of the whole plaque (red < 40)  Percentage of low echogenicity under the surface (red < 30)  Percentage of low echogenicity under the surface (red < 20)  Percentage of low echogenicity of the whole plaque (red < 20)  Percentage of low echogenicity of the whole plaque (red < 30) | 0.48  0.48  0.48  0.48  0.47  0.47  0.46  0.46  0.46  0.46  0.45 | [0.23, 0.73]  [0.23, 0.73]  [0.23, 0.74]  [0.24, 0.73]  [0.22, 0.74]  [0.24, 0.72]  [0.22, 0.73]  [0.22, 0.71]  [0.22, 0.71]  [0.22, 0.71]  [0.20, 0.71] |

AUC, area under the curve; CI, confidence interval
