# Supplementary material for: Diagnostic accuracy of gray-scale analysis on B-mode ultrasound for identifying intraplaque hemorrhage and lipid-rich necrotic core in carotid plaques
Source: Vasc Med. 2026 Feb 25;31(2):175–84. doi: 10.1177/1358863X251410527 (PMC13109598; doi:10.1177/1358863X251410527)
Supplement: sj-docx-3-vmj-10.1177_1358863X251410527 – Supplemental material for Diagnostic accuracy of gray-scale analysis on B-mode ultrasound for identifying intraplaque hemorrhage and lipid-rich necrotic core in carotid plaques [file sj-docx-3-vmj-10.1177_1358863X251410527.docx]

**Collaborators**

**BIOPLAQUE Investigators:** Leo Bonati^1,2^, Gian Marco De Marchis^1,3^, Stefan T Engelter^1,4^, Joachim Fladt^1^, Henrik Gensicke^1,4^, Andrea S Jauslin^1^, Josefin E Kaufmann^1,4^, Philippe Lyrer^1^, Sasha Mukhija^1^, Christopher Traenka^1,4^, Benjamin Wagner^1^, Annaelle Zietz^1^, Tolga D Dittrich^3^, Andrea Wiencierz^5^, Marios Psychogios^6^, Ioannis Tsogkas^6^, Daniel Staub^7^, Edin Mujagic^8^, Thomas Wolff^8^, Mandy D Müller^9^, Mohamed Kassem^10,11^, M Eline Kooi^10,11^, Nils Peters^12^

**Affiliations:**

1. Department of Neurology and Stroke Center, University Hospital Basel and University of Basel, Basel, Switzerland
2. Research Department, Reha Rheinfelden, Rheinfelden, Switzerland
3. Department of Neurology and Stroke Center, University Teaching and Research Hospital, Health Eastern Switzerland (HOCH), Cantonal Hospital St. Gallen, St. Gallen, Switzerland
4. Department of Rehabilitation and Neurology, University Department of Geriatric Medicine FELIX PLATTER, University of Basel, Basel, Switzerland
5. Clinical Trial Unit, University Hospital Basel and University of Basel, Basel, Switzerland
6. Department of Diagnostic and Interventional Neuroradiology, University Hospital Basel and University of Basel, Basel, Switzerland
7. Vascular Medicine/Angiology, University Hospital Basel and University of Basel, Basel, Switzerland
8. Department of Vascular Surgery, University Hospital Basel and University of Basel, Basel, Switzerland
9. Department of Neurosurgery, Inselspital, Bern University Hospital, University of Bern, Bern, Switzerland
10. Cardiovascular Research Institute Maastricht (CARIM), Maastricht University, Maastricht, The Netherlands
11. Department of Radiology and Nuclear Medicine, Maastricht University Medical Center+ (MUMC), Maastricht, The Netherlands
12. Stroke Center, Klinik Hirslanden Zurich, Zurich, Switzerland
